# Students’ and junior doctors’ perspectives on radiology education in medical school: a qualitative study in the Netherlands

**DOI:** 10.1186/s12909-024-05460-9

**Published:** 2024-04-30

**Authors:** Frederike S. Harthoorn, Sascha W. J. Scharenborg, Monique Brink, Liesbeth Peters-Bax, Dylan J. H. A. Henssen

**Affiliations:** 1 Radboud University Nijmegen, Nijmegen, The Netherlands; 2 Department of Medical Imaging, Radboud University Medical Center, Nijmegen, The Netherlands

**Keywords:** Radiology Education, Medical School Curriculum, Learning objectives, Students’ perspectives

## Abstract

**Background:**

Modern medicine becomes more dependent on radiologic imaging techniques. Over the past decade, radiology has also gained more attention in the medical curricula. However, little is known with regard to students’ perspectives on this subject. Therefore, this study aims to gain insight into the thoughts and ideas of medical students and junior doctors on radiology education in medical curricula.

**Methods:**

A qualitative, descriptive study was carried out at one medical university in the Netherlands. Participants were recruited on social media and were interviewed following a predefined topic list. The constant comparative method was applied in order to include new questions when unexpected topics arose during the interviews. All interviews were transcribed verbatim and coded. Codes were organized into categories and themes by discussion between researchers.

**Results:**

Fifteen participants (nine junior doctors and six students) agreed to join. From the coded interviews, four themes derived from fifteen categories arose: (1) The added value of radiology education in medical curricula, (2) Indispensable knowledge on radiology, (3) Organization of radiology education and (4) Promising educational innovations for the radiology curriculum.

**Conclusion:**

This study suggests that medical students and junior doctors value radiology education. It provides insights in educational topics and forms for educational improvement for radiology educators.

**Supplementary Information:**

The online version contains supplementary material available at 10.1186/s12909-024-05460-9.

## Key points

• Ultrasound was suggested to integrate within radiology education in medical curricula.

• Integration of applied radiology in a longitudinal learning community could be explored.

• Regardless of their personal interests, participants valued radiology education in medical curricula.

**Supplementary Information:**

The online version contains supplementary material available at 10.1186/s12909-024-05460-9.

## Background

Can you imagine practicing medicine without using radiologic imaging techniques, such as chest radiographs, CT-scans or ultrasound? It would be almost inconceivable in modern medicine [1]. Coherent to this increasing role of radiology in healthcare, education of radiology in medical curricula has been a topic of discussion with proponents among both clinicians and students for more radiology education [1–9]. The results of a recent review on the role of radiology in medical student teaching reflect this, showing a significant increase in medical articles published over the past decade [10].

Regarding the learning objectives of radiology education, not only consensus between radiologists and clinicians is needed [11–14], but also between students and these groups [9, 15, 16]. The studies of Subramaniam et al. were the only ones that investigated the opinions of clinicians and medical students in a study of three papers to create an overview of the opinions of the different groups on this subject [15, 17, 18]. When focusing on students’ opinions regarding radiology education, various studies investigated these by use of surveys with closed-ended questions [9] and open-ended questions [15, 16]. These studies reported that students recognized the importance of radiology as an educational topic, especially with regard to reading radiographs and the detection of gross abnormalities on medical images [15, 16]. Additionally, students in these studies also stated to have little knowledge regarding the possible health effects of ionizing radiation and MRI safety [9]. Although interesting, these results need further clarification: what exactly do students expect by “reading radiographs”, what pathologies should we consider “gross abnormalities” and how should we teach these subjects to students? To gain deeper insights in quantitative data, a qualitative research method is needed [19]. Therefore, this study sets out to further elucidate students’ perspectives on radiology education in medical curricula by use of individual interviews.

## Methods

### Design

A qualitative, descriptive study with semi-structured interviews was performed. Prior to conducting these interviews, a list of topics was assembled based on relevant scientific literature, discussion sessions between two researchers (F.H. and D.H) and the educational experiences of the research team. Interviews were performed following an inductive iterative process using the constant comparative method [20]. This study was approved by the ethics committee of the Netherlands Association of Medical Education (NVMO, case number 2023.2.9).

### Participants

Master’s students from the Radboud University Nijmegen, The Netherlands, and junior doctors were recruited between August 2020 and October 2020 by placing public advertisements on social media including electronic student environments and Facebook and by contacting students personally, to reach as many students as possible. These ways of recruitment was since no other ways were facilitated. To be included in this study, students needed to be enrolled in the master’s Medicine program of the Radboud University Nijmegen, implying that they had at least some clinical experience. Furthermore, students who followed an elective internship in Radiology were encouraged to participate to gain insights in their experience of an internship.

### Data collection

Interviewees participated in one-on-one semi-structured interviews which were conducted in person, via electronic telecommunication software (e.g. Skype version 8.65.0.78; Skype Technologies, Luxembourg City, Luxembourg Palo Alto, CA, United States ) or by telephone with one of the researchers (F.H.). Semistructured interviews were conducted to obtain nuanced descriptions and extensive, salient data regarding the perspectives on radiology education. The interview schedule was derived from literature-dependent topics and discussions between the researchers. This resulted in a predefined topic list.

During the interviews, participants were encouraged to speak openly about their thoughts and considerations on the subject using open-ended questions. Therefore, it was highlighted that the interviewer had no relations with the board of examiners, the university medical center educational board or any other educational management team.

To ensure reliable data, all interviews were audio- and/or video-recorded, facilitating the transcription of these interviews verbatim afterwards. Prior to the interview, informed consent was obtained from all participants. The transcription of the interviews immediately started after the first interview. When information saturation occurred, two additional interviews were held to control data saturation. When it was confirmed that saturation was achieved, no new subjects were included as this would not result in new insights.

### Data analysis

The transcribed data was thereafter analyzed via direct content analysis [21]. The inductive iterative process was performed using the constant comparative method. Data analysis started after completion of the first interview. Codes derived from the previous interview were used as starting point for coding the next one and additional codes were added when needed. Two researchers (F.H. and D.H) analyzed four interviews independently in order to compare the coding process. Discrepancies in coding were solved by discussion and concession. Thereafter, one researcher (F.H.) coded the remaining interviews. The coding process was performed using Atlas.ti software, version 8.2.29.0 (ATLAS.ti Scientific Software Development GmbH, Berlin, Germany). As a result, the created coding list was used to make an overview of categories and themes as a final product.

## Results

Sixteen subjects responded to the recruitment, one student was excluded due to not yet being enrolled in the master’s program, resulting in a total of fifteen participants who were interviewed. Tables 1 and 2 give an overview of the characteristics of the participants. All answers were collected via interviews; nine via Skype, one via Facetime, three via telephone and two in person. The interviews lasted between 25 and 50 min.

**Fig. 1:**
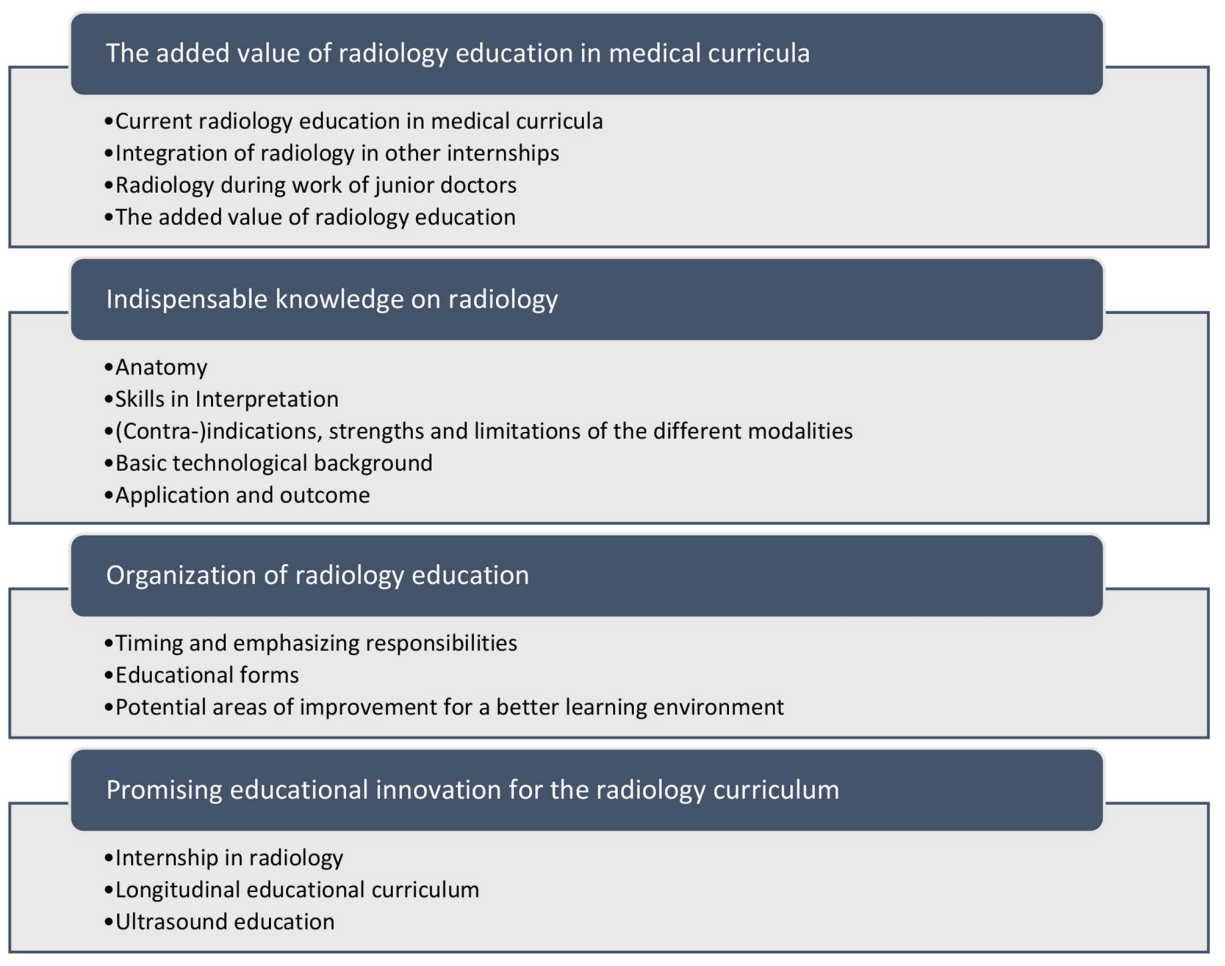
Summary of students’ perspectives on radiology education in medical curricula organized in themes and categories

Four themes derived from fifteen categories arose from the qualitative data: (1) the added value of radiology education in medical curricula, (2) indispensable knowledge on radiology, (3) organization of radiology education and (4) promising educational innovations for the radiology curriculum (Fig. 1).

**Table 1:** Characteristics of the participants

Characteristics	*All (n = 15)*	
	*n*	%
**Gender**		
Male	4	*26.67%*
Female	11	*73.33%*
**Educational status**		
1^st^ year of Master’s Phase	1	*6.67%*
2^nd^ year of Master’s Phase	2	*13.33%*
3^rd^ year of Master’s Phase	3	*20.00%*
Graduated	9	*60.00%*
**Specialty of employment**		
Internship/student	6	*46.67%*
Emergency Medicine	1	*6.67%*
General Practitioner	1	*6.67%*
Geriatrics	1	*6.67%*
Intensive Care	1	*6.67%*
Pediatrics	1	*6.67%*
Psychiatry	1	*6.67%*
Surgery	1	*6.67%*
Insurance	1	*6.67%*
Post graduate, no job	1	*6.67%*
**Attended an elective internship in Radiology**		
Yes	8	*53.33%*
No	7	*46.67%*

**Table 2:** Relation between educational status and specialty of employment with participation in the elective internship of Radiology

	Participation in elective internship of Radiology			
	Yes (*n*)	No (*n*)	Participation (%)	
**Educational status**	**1**	**5**	**33.33**	
*1st year of Master’s Phase*	*0*	*2*	*0.00*	
*2nd year of Master’s Phase*	*0*	*2*	*0.00*	
*3rd year of Master’s Phase*	*1*	*1*	*66.67*	
**Graduated; specialty of employment**	**7**	***2***	**66.67**	
*Emergency Medicine*	*1*	*0*	*100.00*	
*General Practitioner*	*1*	*0*	*100.00*	
*Geriatrics*	*1*	*0*	*100.00*	
*Intensive Care*	*0*	*1*	*0.00*	
*Pediatrics*	*1*	*0*	*100.00*	
*Psychiatry*	*1*	*0*	*50.00*	
*Surgery*	*1*	*0*	*100.00*	
*Insurance*	*0*	*1*	*0.00*	
*Post graduate, no job*	*1*	*0*	*100.00*	
***Years working as a junior doctor***				
*0–1 year*	*3*	*2*	*60.00*	
*1–2 years*	*2*	*0*	*100.00*	
*2–3 years*	*1*	*0*	*100.00*	
*3–4 years*	*1*	*0*	*100.00*	
***Total***	***8***	***7***	***53.33***	

### The added value of radiology education in medical curricula

#### ***Current radiology education in medical curricula***

Interviewees expressed a heterogeneous exposure to radiology education moments, due to their preferences (e.g. attending elective courses) and changes in the medical curriculum. However, all interviewees stated that they received education on interpretating chest radiographs. Despite that, the medical curriculum paid little attention to it and interviewees felt ill-prepared to perform this task adequately. Additionally, participants expressed that systematic reading of chest radiographs, once taught, was easily forgotten due to a lack of repetition.

Interviewees considered radiology education fragmented throughout the study program and lacking proper structure. Participants also believed that radiological images were rather used as a tool to support other educational moments and indicated that they were often not aware of the relevance of gaining knowledge in radiology.

#### ***Integration of radiology in other internships***

 Participants expressed greater exposure to radiological images during their internships compared to their theoretical courses. Nonetheless, most interviewees experienced little or no expectations from supervisors regarding radiologic knowledge. Therefore, almost all their radiologic knowledge was acquired via self-study and critical evaluation of radiologic knowledge by an expert was lacking. Participants expressed that they did master different skills, dependent on their clinical exposure during the internships. This includes balancing pros and cons when choosing a radiologic exam, how to write a decent question for the radiologist and systematically reviewing chest radiographs.

#### ***Radiology during the work of junior doctors***

 Although the exposure of radiology varied in the participants’ jobs, several aspects of knowledge in radiology were described as advantageous for their work. For example: understanding and interpretating a radiologic report or conclusion, knowledge in different imaging techniques and useful skills for requesting a radiologic exam. This knowledge was considered important for night shifts, when junior doctors have little supervision. Participants appointed that this knowledge was gained via clinical practice and experiences and not through received education.

#### ***The added value of radiology education***

There were no opponents for radiology education among the interviewees. The majority believed that knowledge in radiology would be beneficial for all medical students as radiology is an omnipresent, important diagnostic tool in medical disciplines. Therefore, they considered it important to integrate into medical curricula.

One participant questioned if other disciplines deserve more time in the already crowded medical curricula instead of radiology. Several participants expressed that specific radiologic knowledge for certain specialisms should be gained during residency. However, they dismissed this consideration since the wide occurrence of radiology in various specialisms is also the reason that basic knowledge in radiology would be beneficial for (almost) all medical students. Consequently, participants experienced a need for more education in the basics of radiology.

*“For me [as a medical advisor for insurance] it is not that relevant anymore to know all that. But yes, most of the students will obviously work in the clinical sector or will end up in the treatment sector” – Junior doctor*.

### Indispensable knowledge on Radiology

#### ***Anatomy***

Knowledge of anatomy was considered of great importance in order to understand a radiologic image and to distinguish normal images from abnormal ones. CT-scans and radiographs were thought to be imaging techniques on which students should be able to recognize anatomy. Whether the same applies to MRI was a point of discussion, because of the complexity of the imaging technique itself.

*“If you do not understand the anatomy, you will not understand the image and vice versa […] so you will not be able to assess an image without knowing the anatomy” – Student*.

#### ***Skills in interpretation***

The interpretation of chest radiographs was considered a potential learning topic, but discussion arose to what extent this topic should be taught. Beliefs varied from interpretating the whole radiologic image with an own conclusion, to only systematically reviewing, to questioning if this should be taught at all during medical school. Interpretation of other imaging modalities (i.e. MRI and CT) was seen as a specialistic skill that should not be a learning goal in medical curricula. However, opinions differed as to which depth a student should be able to recognize certain anatomical landmarks and/or abnormalities.

**Fig. 2:**
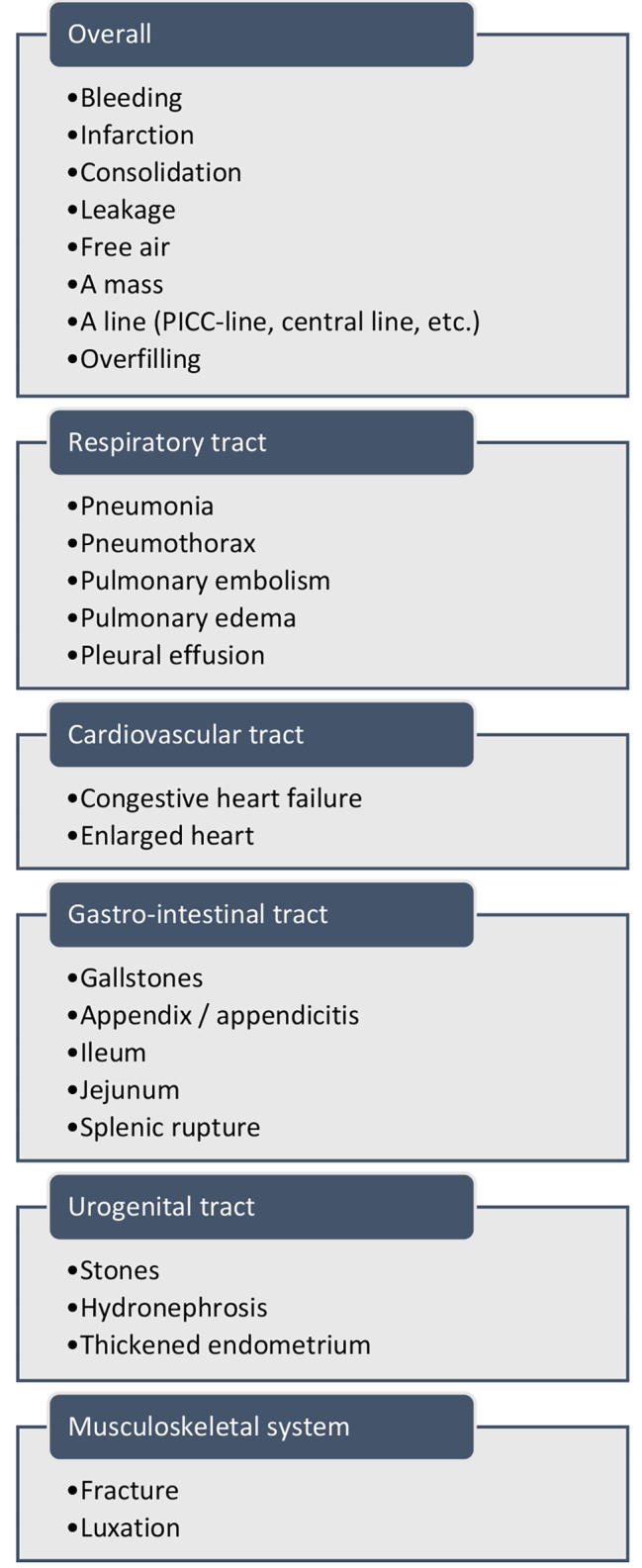
A list of mentioned structures or 1 abnormalities that junior doctors should recognize according to the respondents

Overall, it was considered important that students can differentiate normal from abnormal whilst looking at a radiologic image. Furthermore, participants indicated the importance of recognizing the most prevalent anomalies on the most commonly used modalities (Fig. 2) and the abnormalities that need rapid medical intervention. Two frequently given examples were recognizing fractures and pneumonia on (chest)radiographs.

*“…I believe that you should be able to assess the acute pathologies of every modality. This enables you to get ahead in the clinical decision-making process, when no radiologist is present on short notice” – Junior Doctor*.

#### ***Basic technological background***

Participants expressed that basic technological background of radiological images should be less prominent in medical curricula. It was experienced that there is too much focus on these theoretical aspects, which are too specific for junior doctors. However, interviewees did indicate that a certain (basic) knowledge is required to understand an image.

*“You need basic understanding of how the modality works…At the beginning of the curriculum, our education focuses mainly on the different techniques. I just miss the clinical application” – Student*.

#### ***(Contra-)indications, strengths and limitations of the different modalities***

Knowledge of (contra-)indications of the different techniques was considered important by the majority of the interviewees. They believed it to be an important part of the clinical reasoning process. Additionally, differentiation in indication between available techniques such as CT, MRI or ultrasound was believed to be important.

*“…it is paramount to learn the most important indications for the different radiological examinations for the most frequently encountered pathologies during every internship” – Junior Doctor*.

These thoughts were accompanied by the idea that a student must know the strengths and limitations of commonly used radiologic exams. Knowledge on radiation, patient characteristics, influence of timing on accuracy of an image, sensitivity and specificity and false-positivity and false-negativity were suggested. There was a discrepancy in whether this is essential to teach or just good to know between participants.

*“Knowing not to order an ultrasound for a heavily obese patient” – Student*.

#### ***Application and outcome***

The application of radiologic studies was considered an important educational topic. This included the added value of implications and consequences of the outcome of a radiologic study, the costs of different modalities, knowing which tests are available in specific circumstances and knowing what to mention when requesting a radiologic study.

*Radiological imaging is getting better, fancier and clearer, but consequently it also getting more expensive. …When I believe that it is important to know something, I need to consider whether it will change my course of action for a patient. Only then, I must order the radiological examination – Junior Doctor*.

Knowledge in the use of outcome of a study was regarded to be important as well. This included items such as understanding the terminology, looking critically at the conclusion and the role as a clinician to create a link between the clinical case and the image.

Lastly the role of the radiologists was mentioned. Interviewees believed that the ability to consult a radiologist should always be present when in doubt, for both application and outcome. Additionally, it was found important to create more insight into the tasks of a radiologist, so it would become clearer what can be asked and expected, and what is important information to provide when requesting a radiologic examination.

*“That would be very interesting indeed, to know what the radiologist considers important regarding an application. I do not know that at all actually. I write down what the symptoms are and what diseases I am suspicious of, but I am not sure whether this is actually knowledge the radiologist needs. I can imagine that there is a lot to gain in that area” – Junior Doctor*.

### Organization of Radiology Education

#### ***Timing and emphasizing responsibilities***

It was believed that radiology education would be more useful if taught in the master’s phase, since students would be able to understand the value of this knowledge in a clinical context. Furthermore, interviewees believed repetition to be the key for both creating a better learning environment and ensuring less time investment in the overcrowded medical curriculum. Some participants suggested an integrated radiology curriculum including only basic topics while others advised against a separate radiology course.

Accompanied by this view, it was believed that radiology education should be integrated within other internships. Interviewees suggested teaching specific modalities before the start of different internships. For example, formal education on how to read radiological examinations of the brain (i.e. MRI and/or CT) should be organized prior to neurology rotations and principles of ultrasound should be taught prior to the gynecology internship. Education on indications and application was suggested to be taught during the last year of the master’s phase. This is because in the Netherlands a medical student is only allowed to perform this task during this last phase of the master. Recapitulating some radiology teaching material prior to starting the elective internships was also suggested.

*“Before starting my surgery internship, I wanted to have some education about reading radiographs of fractures. We did receive some education on this topic, but it was really short, and it was not really about radiology. That might be a good addition.” – Student*.

#### ***Educational forms***

Participants suggested an integrated, repetitive radiology curriculum within the courses and internships of other specialties. Within this curriculum, students would like to see applied radiology and applied anatomy integrated in clinical cases, as well as a combination between self-study and practice. Other suggested teaching forms were working groups, computer guided education, e-learnings, self-study assessments, education in the dissection room and radiology meetings.

*“I believe that it would be best if radiology education is integrated in the education preparing a student for a specific internship: these are the investigations that you will see encounter during this internship.” – Junior Doctor*.

Although digital education (e-learnings and computer-guided education) was considered a good tool to learn recognizing images and to teach the basics of radiology, interviewees also had a negative view towards these teaching forms. They highlighted the pitfalls of having no feedback or possibility to ask questions, resulting in a passive learning style.

*“I consider e-learnings useful to learn about the basics, but I think lectures or small group assignments are more useful for clinical discussions as you can have interaction with a professional.” – Student*.

**Fig. 3:**
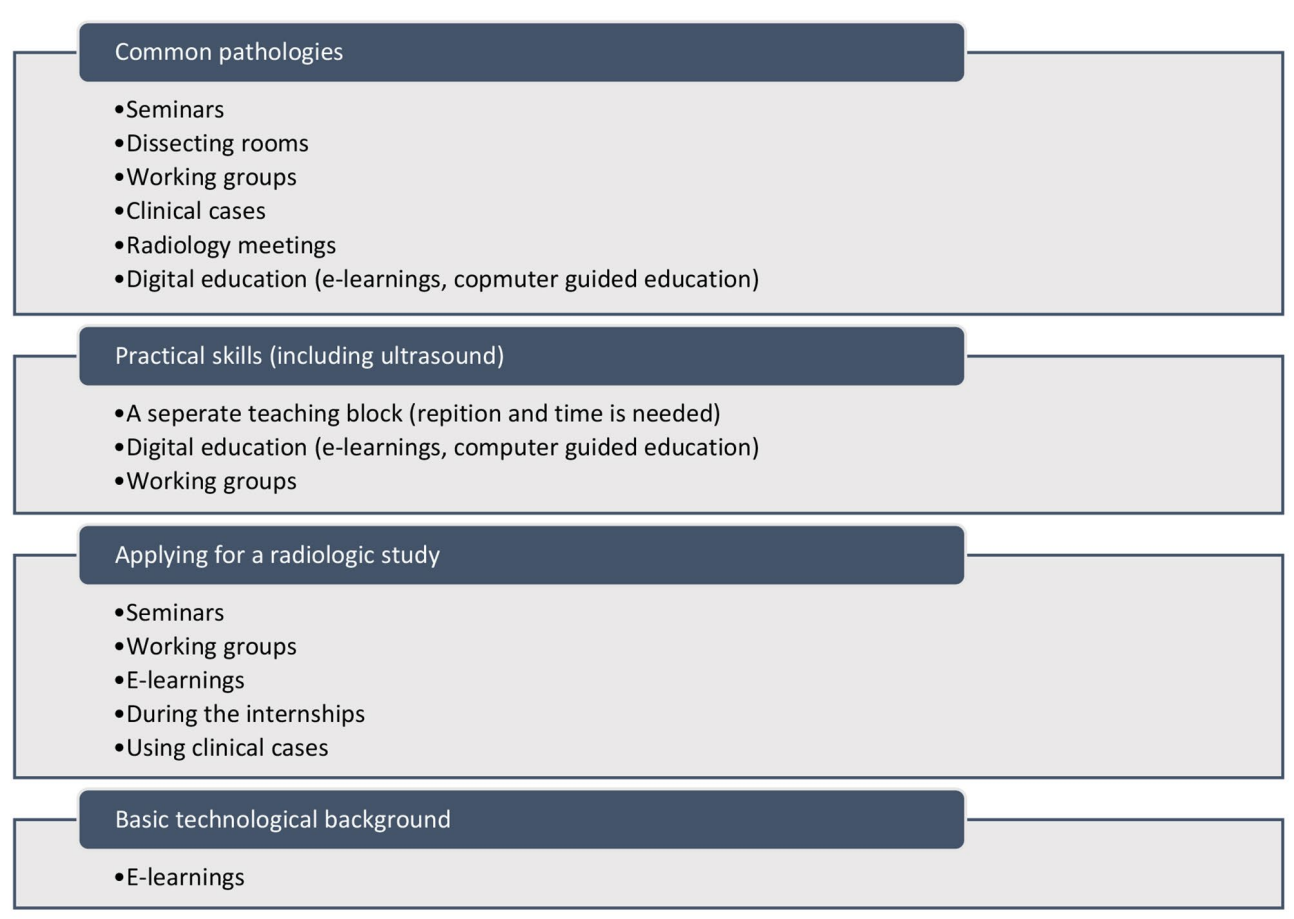
Suggested educational forms per educational topic

The majority of the participants had a positive view towards interactive education forms. In Fig. 3 an overview of the proposed educational forms per educational topic is shown. Additionally, the importance of having good references present was highlighted.

*“It is better to use radiologic images that are examples of the really obvious during medical education. Usually, you will encounter complicated pathologies and as a beginning intern you need to study more simple pathologies, for example pneumonia on chest radiographs.” – Student*.

#### ***Potential areas of improvement for a better learning environment***

Interviewees believed that teaching radiology before starting a new internship would be beneficial to their radiologic knowledge and could result in increased self-esteem. Linking radiology education to a specific specialty and aligning the education with clinical practice was considered important. It was believed essential that students understand why certain topics are discussed. Other suggestions to enhance the learning environment were: enough exposure and repetition, providing feedback on questions in e-learnings and aligning learning objectives with the changing tasks of students during the years of their internships.

*“You will have to implement your knowledge right after learning a radiological principle. Thereby, you will use the acquired knowledge which will result in better recall” – Student*.

### Promising educational innovation for the radiology curriculum

#### ***Internship in radiology***

While some participants suggested a regular (mini-)internship in radiology, the majority of the interviewees believed an elective internship in radiology to be better for personal deepening, also including the student’s personal learning objectives. Integration of a couple (internship)days of radiology in regular internships was also proposed.

However, if an internship would be created, participants suggested a duration of two weeks with integrated educational moments. The educational objectives of the internship were difficult to come up with and ideas on timing in the medical curriculum differed from before the start of the regular internships to the last year. By providing the internship in the first master year, the gained knowledge would come in handy during the next internships. On the other hand, there would be a danger that students would not understand the importance of this knowledge and could potentially have too little foreknowledge to help them assess and interpret the images.

#### ***Longitudinal educational curriculum***

The idea of a longitudinal learning community (LLC) was pitched among participants as a new type of radiology education in the medical curriculum. This was described as a community-based approach to learning that stimulates meaningful student interaction via repetitive small-group learning and peer-group evaluation during a time period of more than one year. All participants were advocates for the suggested LLC and believed it would be a great addition to the current medical curriculum. One student suggested facilitating the LLC and withdrawing the internship in radiology. The aforementioned topics were proposed to be incorporated in the LLC.

*“I think that it is a really good idea to have a continuous process of learning activities which helps you to expand your knowledge. And indeed, radiological imaging is somewhat different for different internships as some techniques are more often used than others in different settings” – Junior Doctor*.

It was believed that the LCC would increase the attention for radiology education during other internships. It was noted that the complexity of the study materials taught in the LLC could progress over time. Although, it was disclosed that fragmentation could also be a pitfall of an LLC in radiology.

*“You could embed an afternoon or day of radiology into other internships. So that during the neurology internship, you will observe the work of the neuro-radiologist. Then you will have more exposure.” – Student*.

#### ***Ultrasound education***

Interviewees noted that ultrasound is an emerging modality and believed education in ultrasound to be attractive. Discussions arose on both the added value of the theoretical aspects (including interpretation) as well as the practical aspects of ultrasound education.

*“Good ultrasound education is lacking in the medical curriculum. Whereas ultrasound, in my opinion, is the bedside diagnostic tool of the future, especially with the hand-held ultrasounds which fit in your pocket.” – Student*.

For ultrasound education to become useful, participants believed repetition and basic educational topics to be essential components. It was believed that these topics should be the same as other radiologic modalities. Regions that were suggested to teach were the abdomen and heart. As for teaching forms it was suggested to either use a separate course consisting of a combination of computer-guided education and self-study or integrate the study material via clinical cases (applied radiology).

However, there was disagreement on teaching practical sonography skills. Opponents mainly stated that this would be too specialized, while an advocate highlighted that it would be beneficial to master this skill since ultrasound is a dynamic examination.

*“Performing an ultrasound examination properly and interpreting those images adequately, that is not something you will learn in one group session. For that, you would really need more time in the medical curriculum, to teach it consequently”- Student*.

## Discussion

This study suggests that medical students and junior doctors value radiology education in medical curricula as they see it as a relevant topic, regardless of personal interests. However, radiology education in its current form was criticized. Adaptions to facilitate a more integrated and applied form of radiology education were suggested, in order to establish the skills a junior doctor should master. An elective radiology internship was suggested for those more interested.

Our results are in line with outcomes reported by Subramaniam and colleagues [15]. They found that medical students considered (1) *learning to systematically analyze* radiographs, (2) distinguishing normal from abnormal and (3) identifying gross abnormalities important learning goals. However, several new topics arose from our study. For example, we found that students believe that less time should be invested in the theoretical background of the radiologic techniques. Nevertheless, in a previous study, students also reported the lowest mean score for “Basic knowledge of radiation protection, including timing of organogenesis and radiation effects”. This indicates that students regard this learning goal least interesting [15]. In our study, on the other hand, the students detail their comments and provide insights on how to implement this theoretical aspect of radiology in daily educational practice; For example by in-time learning and using clinical cases to teach radiology in a more applied way. In addition, our study suggested that students have nuanced views on the depth of their knowledge with regard to different topics. Also, students suggest in this paper that more time should be created for learning applied radiology, and that ultrasound education should be implemented more broadly. Nevertheless, this study was unable to investigate the nuanced views of students and junior doctors on each topic. Regarding the basic technological background, further research should aim to provide a detailed overview of the benefits and limitations as perceived by students and junior doctors regarding this subject. Such a detailed overview of the topics described in this study could help to further shape radiology education of the future.

Participants suggested integrating ultrasound as an imaging technique within the medical curriculum. Discussion arose if this should also include practical skills. This increased interest in ultrasound education has been shown in literature [22, 23]. Although the theoretical aspect of this technology is getting more implemented in medical curricula in Europe, and ultrasonography is practically taught in some countries, not all universities have developed an ultrasound curriculum to teach the practical skills [24]. One explanation that could help understand why such an ultrasound curriculum is not ubiquitous within modern medical curricula, could be that a well-designed ultrasound curriculum is needed for optimal integration that meets students’ expectations and matches the existing clinical needs. Nonetheless, a recent study did provide recommendations for such an ultrasound curriculum in medical school [25], although further investigation of the learning outcomes would be paramount [26–29]. Positive outcomes on teaching anatomy have been reported, though the impact of ultrasound education on clinical examination skills of medical students is less clear and needs further investigation [29, 30].

The possibility of developing an LLC to improve radiology curricula was positively reviewed by participants. Literature showed examples of national radiology curricula that were developed in the UK, Australia and Germany [23, 31]. However, it was unclear whether these curricula are also integrated in medical curricula. To our knowledge no national LLC curriculum has yet been developed in the Netherlands. Since positive outcomes have been reported of an integrated imaging curriculum [23, 32], we believe it would be beneficial to explore this educational form of radiology education. It has been reported that a vertically integrated “virtual” radiology internship is as effective as a freestanding internship [33]. The remark of the interviewees that a radiology internship should be elective when a LLC is applied is in line with these findings.

This study has several strengths and limitations. The qualitative research design provided a detailed insight into students’ perspectives on radiology education in medical curricula. By including junior doctors, more insight into the gaps of the current radiology education and the challenges of radiology in daily clinical work was obtained. The group of participants varied in personal interests and interest in future specialties, which was essential to get insight into whether every medical student would benefit from radiology education, and which topics should be taught. Lastly, some participants followed an elective internship in radiology and others did not. This information was used to obtain more information if an internship in radiology would be useful, either mandatory or elective.

The generalizability of these data is affected by the small group of included participants. This should be seen as a limitation of this study. Second, representativeness could have been affected by the method of recruiting students and junior doctors, creating selection bias. Third, only students from one University Centre in the east of the Netherlands were included, potentially creating responses that are not representative for students participating in other Dutch curricula. Finally, the fact that only junior doctors were included might have led to unbalanced answers when it comes to professional needs later in their medical career and to the costs of suggested improvements. Although we have no reason to assume that the interviewees cannot reflect the large group of medical students, more qualitative research on the importance of radiology education is warranted to confirm the presented findings.

## Conclusion

This study suggests that medical students and junior doctors consider radiology education important within medical school curricula and provided insight into educational topics and ways to improve the current curriculum. Participants were positive about an integrated radiology curriculum that included applied radiology and incorporating more ultrasound education in the medical curriculum. The implementation of a LLC in radiology, incorporating ultrasound education, could be investigated. Overall, more research is needed to get information from more students on these specific subjects and get an agreement between clinicians and students on these topics.

### Electronic supplementary material

Below is the link to the electronic supplementary material.

Supplementary Material 1

## Data Availability

The dataset which was generated from the interviews and analyzed during the current study is not publicly available since individual privacy could potentially be compromised. Data are however available from the corresponding author on reasonable request.
