# Supplementary material for: Students’ and junior doctors’ perspectives on radiology education in medical school: a qualitative study in the Netherlands
Source: BMC Med Educ. 2024 Apr 30;24:479. doi: 10.1186/s12909-024-05460-9 (PMC11062010; doi:10.1186/s12909-024-05460-9)
Supplement: Supplementary file 1 — Supplementary Material 1 [file 12909_2024_5460_MOESM1_ESM.docx]

| **Part A** | |
| --- | --- |
| Objective: To ascertain the depth of knowledge possessed by a medical student/graduate regarding the domains of Radiology and Nuclear Medicine. | |
| ***Sub questions*** | - *What knowledge do you possess regarding Radiology and Nuclear Medicine?* - *How do you apply this knowledge in your daily activities?* |
|  | |
| **Part B** | |
| Objective: To ascertain the desired foundational knowledge in the fields of Radiology and Nuclear Medicine that a physician should possess upon completion of the Medicine training program. | |
| ***Sub questions*** | - *What is considered fundamental knowledge regarding Radiology and Nuclear Medicine?* - *How do students perceive their own knowledge regarding Radiology and Nuclear Medicine in light of their daily activities?* - *What knowledge do students anticipate to have upon completion of their medical education with regard to the fields of Radiology and Nuclear Medicine?* |
|  | |
| **Part C** | |
| Objective: To ascertain the methods of teaching Radiology and Nuclear Medicine in medical curricula and to probe views and expectations of students with regard to this. | |
| ***Sub questions*** | - *In what manner do students acquire knowledge about Radiology and Nuclear Medicine within medical curricula?* - *What types of education are provided?* - *What topics are covered in this education?* - *Are there various options for education, such as mandatory or optional courses?* - *Is participation in clinical internships based on individual choice, or is it required for everyone?* - *How do the students perceive the amount and level of detail of education on Radiology and Nuclear Medicine within the program?* - *Is there any specific education on Radiology and Nuclear Medicine that students feel is missing, inadequate or superfluous?* |

**Supplement 1. Interview guide**
